# Generation of a luciferase-based reporter for CHH and CG DNA methylation in *Arabidopsis thaliana*

**DOI:** 10.1186/1758-907X-4-1

**Published:** 2013-04-05

**Authors:** Thanh Theresa Dinh, Michael O’Leary, So Youn Won, Shengben Li, Lorena Arroyo, Xigang Liu, Andrew Defries, Binglian Zheng, Sean R Cutler, Xuemei Chen

**Affiliations:** 1 Department of Botany and Plant Sciences, Institute of Integrative Genome Biology, University of California, Riverside, CA, 92521, USA; 2 NSF ChemGen IGERT program, University of California, Riverside, CA, 92521, USA; 3 Howard Hughes Medical Institute, University of California, Riverside, CA, 92521, USA; 4 Department of Plant Pathology, University of California, Davis, CA, 95616, USA; 5 State Key Laboratory of Genetic Engineering and Institute of Plant Biology, School of Life Sciences, Fudan University, Shanghai, 200433, China

**Keywords:** Luciferase, RdDM, DNA methylation, *MET1*, *AGO4*, *DRM2*, Methotrexate

## Abstract

**Background:**

DNA methylation ensures genome integrity and regulates gene expression in diverse eukaryotes. In *Arabidopsis*, methylation occurs in three sequence contexts: CG, CHG and CHH. The initial establishment of DNA methylation at all three sequence contexts occurs through a process known as RNA-directed DNA methylation (RdDM), in which small RNAs bound by Argonaute4 (AGO4) guide DNA methylation at homologous loci through the *de novo* methyltransferase DRM2. Once established, DNA methylation at each of the three sequence contexts is maintained through different mechanisms. Although some players involved in RdDM and maintenance methylation have been identified, the underlying molecular mechanisms are not fully understood. To aid the comprehensive identification of players in DNA methylation, we generated a transgenic reporter system that permits genetic and chemical genetic screens in *Arabidopsis*.

**Results:**

A dual *35S* promoter (*d35S*) driven luciferase (*LUC*) reporter was introduced into *Arabidopsis* and *LUCL*, a line with a low basal level of luciferase activity, was obtained. *LUCL* was found to be a multi-copy, single-insertion transgene that contains methylated cytosines in CG, CHG and CHH contexts, with the highest methylation in the CG context. Methylation was present throughout the promoter and *LUC* coding region. Treatment with an inhibitor of cytosine methylation de-repressed luciferase activity. A mutation in *MET1*, which encodes the CG maintenance methyltransferase, drastically reduced CG methylation and de-repressed *LUC* expression. Mutations in *AGO4* and *DRM2* also de-repressed *LUC* expression, albeit to a smaller extent than loss of *MET1*. Using *LUCL* as a reporter line, we performed a chemical screen for compounds that de-repress *LUC* expression, and identified a chemical, methotrexate, known to be involved in biogenesis of the methyl donor.

**Conclusion:**

We developed a luciferase-based reporter system, *LUCL*, which reports both RdDM and CG maintenance methylation in *Arabidopsis*. The low basal level of *LUCL* expression provides an easy readout in genetic and chemical genetic screens that will dissect the mechanisms of RdDM and methylation maintenance.

## Background

An epigenetic modification that influences gene expression and genome stability is cytosine DNA methylation, which involves the addition of a methyl group to the five position of the pyrimidine cytosine. This mark in transposable elements or intergenic regions is often associated with transcriptional gene silencing (TGS) and contributes to genome stability. In *Arabidopsis*, *de novo* methylation is guided by small and long noncoding RNAs and is referred to as RNA-directed DNA methylation (RdDM). The RdDM pathway can be divided into three main components. First, in an unknown manner, RNA polymerase IV (Pol IV) is recruited to target loci and generates single-stranded RNA (ssRNA). Second, the ssRNA is made double-stranded by RNA-Dependent RNA Polymerase 2, and the double-stranded RNA is further processed into 24 nucleotide (nt) siRNAs by DICER-LIKE 3. One strand is loaded into Argonaute4 (AGO4), the major effector protein of 24 nt siRNAs. Third, in parallel, RNA polymerase V (Pol V) is also recruited to these loci by an unknown mechanism and generates long noncoding transcripts. It has been proposed that these transcripts act as a scaffold for the recruitment of the siRNA-AGO4 complex. This further facilitates the recruitment of other downstream effectors such as Involved In *De Novo* 2 and the *de novo* methyltransferase DRM2 to methylate these loci (reviewed in [1]). Although many genes in this pathway have been identified, key outstanding questions on the underlying molecular mechanisms of RdDM remain to be answered.

In *Arabidopsis*, there are three types of cytosine methylation: CG, CHG and CHH. CG and CHG are considered symmetric methylation, whereas CHH methylation is considered asymmetric methylation. The three types of DNA methylation are all established by RdDM, but are maintained via different mechanisms after DNA replication. CHH methylation is maintained by constant *de novo* methylation by DRM2 and other players in the RdDM pathway. CHG methylation is maintained by a reinforced loop between the DNA methyltransferase Chromomethylase3 and histone modifications (reviewed in [1]). CG methylation is maintained by DNA Methyltransferase 1 (MET1) and intriguingly, MET1 has been shown to also be required for full levels of *de novo* methylation of CG dinucleotides [2].

The players involved in CG maintenance methylation are conserved between mammals and plants. Specifically, in mammals, newly replicated DNA is hemi-methylated and DNMT1, the MET1 ortholog in mammals, is responsible for methylating the newly synthesized strand [3]. DNMT1 is recruited to newly replicated DNA through interactions with UHRF1 and PCNA. UHRF1 specifically binds to hemi-methylated CG dinucleotides [4-7], and PCNA is present at the replication fork [8]. In *Arabidopsis*, CG maintenance methylation is mediated by *MET1*[2] and three *Variant In Methylation* genes *(VIM1-3)*, which are orthologs of UHRF1 [9,10]. Like in mammals, the recruitment of VIM1 to hemi-methylated DNA facilitates the recruitment of MET1, which results in the methylation of the daughter strand. In addition, CG maintenance methylation in *Arabidopsis* also requires *Deficient In DNA Methylation 1* (*DDM1*), a chromatin remodeling protein [11,12].

CG methylation is located not only at transposable elements/intergenic regions, but also in gene bodies. About one-third of genes have CG methylation in their coding regions in *Arabidopsis* (this number is higher in mammals), and gene body CG methylation is also maintained by *MET1*[13]. CG methylation in gene bodies does not cause silencing, unlike methylation at transposons [14]. In fact, genes harboring body methylation are moderately to highly expressed [14-16]. The purpose of CG body methylation is still unclear; however, hypotheses on its potential functions include the suppression of cryptic promoters within coding regions [14,17] and the enhancement of accurate splicing [18,19].

Here, we describe the generation of a luciferase (*LUC*)-based reporter line that enables screening for genes involved in CG maintenance methylation as well as CHH methylation via RdDM in *Arabidopsis*. Due to the extensive CG methylation in the *LUC* coding region, the reporter may also help to understand the functions of gene body methylation. This line is named *Luciferase Harboring CG Methylation, Low* (*LUCL*) for its high levels of CG methylation and low levels of *LUC* expression. Consistent with the finding that *LUCL* harbors high levels of CG methylation, the *met1-3* mutation resulted in a release of DNA methylation at the transgene promoter and throughout the *LUC* coding region and drastic de-repression of *LUC* expression. Interestingly, introducing *ago4-6* and *drm2-6* mutations into *LUCL* also resulted in the de-repression in *LUC* expression, thus *LUCL* also reports *de novo* methylation through RdDM, although RdDM contributes to the silencing of *LUCL* to a much lesser extent than CG maintenance methylation. The near complete silencing of *LUC* expression in *LUCL* means facile screens can identify genetic mutations or compounds that release *LUC* silencing. The performance of a chemical genetics screen with *LUCL* led to several hit compounds. One of the hit compounds was methotrexate (MTX), which is known to indirectly prevent the production of S-adenosyl methionine (SAM), the methyl donor [20]. Treatment of plants with MTX resulted in reduced DNA methylation at, and de-repression of, six endogenous RdDM loci that were examined. Therefore, *LUCL* could serve as a great tool to probe the mechanisms of DNA methylation.

## Results and discussion

### Development of the luciferase reporter line, *LUCL*

**Figure 1:**
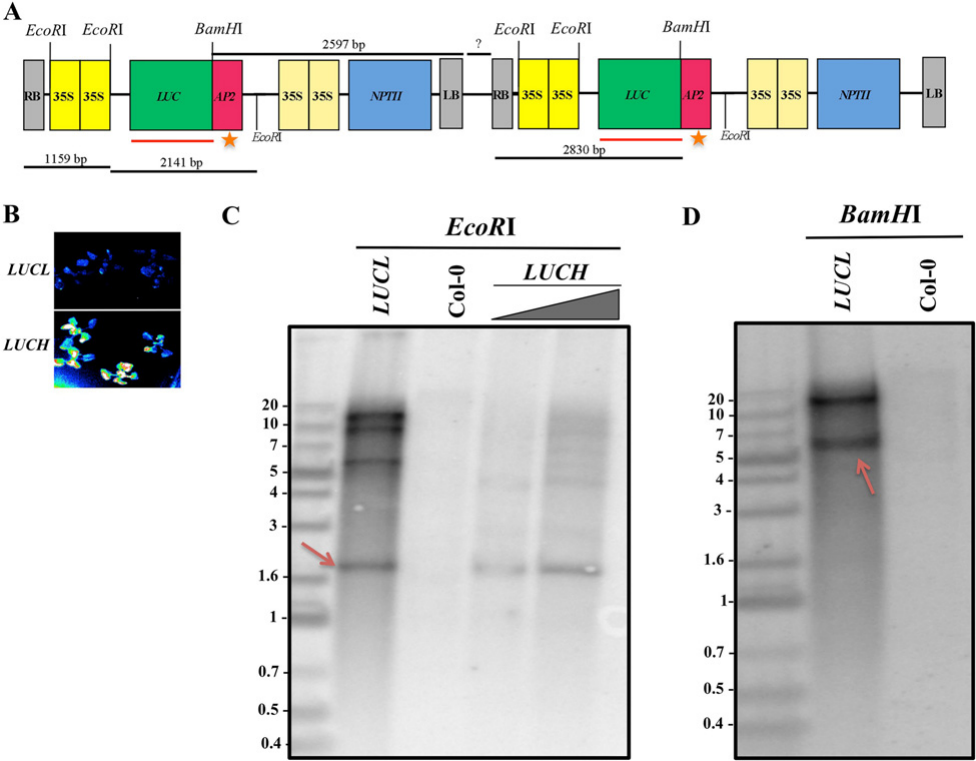
***LUCL*is a multi-copy, single-insertion transgene. (A)***LUCL* as a multi-copy transgene. Only two tandem copies are shown, with each copy extending from RB (right border of the transfer DNA) to LB (left border of the transfer DNA). Restriction sites and distances between sites are noted. The question mark indicates the unknown distance between two tandemly arrayed copies. The stars indicate the miR172 binding sites. The red lines depict the region used as a probe in the Southern blots in (**C**) and (**D**). **(B)** Luciferase luminescence from *LUCL* and *LUCH* seedlings. Ten-day-old seedlings grown on the same plate were imaged for luciferase luminescence using a CCD camera. The blue spots in the *LUCH* sector represent seedlings with luciferase luminescence. The lack of signals in the *LUCL* sectors represents the absence of luciferase luminescence. **(C)** Southern blot analysis of *LUCL*, Col-0 and *LUCH*. The gray triangle indicates increasing amounts of genomic DNA from *LUCH*; the left lane has an amount of DNA equal to *LUCL* whereas the right lane contains twice that of *LUCL.* Genomic DNA was digested with *EcoR*I and hybridized with a probe corresponding to the *LUC* coding region (red line in (A)). The 2.1-kb band corresponding to the *LUC-AP2* fragment is indicated by a red arrow. The intensity of the 2.1-kb band in *LUCL* is much higher than that in *LUCH*. **(D)** Southern blot analysis of *LUCL* and Col-0. Genomic DNA was digested with *BamH*I and hybridized to a probe corresponding to the *LUC* coding region (red line in (**A**)). The approximately 6-kb band (red arrow) represents the possibility of a multi-copy transgene as the distance between the two *BamH*I sites in two tandemly arrayed copies is 5.4 kb (not counting the unknown distance between the LB and RB (question mark)).

Luciferase-based reporters have been used extensively as probes for different biological processes [21-24]. Initially, we aimed to develop a luciferase-based transgene that reports miRNA activity. For this purpose, we generated a transgene in which the *Luciferase* (*LUC*) coding region fused to a portion of the *APETALA2* (*AP2*) gene that contains the miR172 binding site [21] was behind a dual *35S* promoter from the *cauliflower mosaic virus* (Figure 1A). In the same transfer DNA, dual *35S*-driven *Neomycin Phosphotransferase II* (*d35S::NPTII*) served as a selectable marker for plant transformation (Figure 1A). The transgene was introduced into the *RNA-dependent RNA polymerase6-11* (*rdr6-11*) mutant background to prevent post-transcriptional gene silencing [25-27]. We established two independent *Arabidopsis* lines containing this transgene, *LUCH*[21] and *LUCL* (*Luciferase Harboring CG Methylation, Low*), the latter being the topic of this study. Although the transgene in *LUCL* and *LUCH* is identical in sequence, *LUCL* has a much lower level of luciferase activity than *LUCH* (Figure 1B). In fact, the luciferase activity in *LUCL* was practically non-existent and comparable to that of the wild type (Col-0) (Figure 1B).

### *LUCL* is a multi-copy, single-insertion transgene

We first characterized the nature of the transgene insertion in *LUCL* in comparison to *LUCH*. *LUCH* was shown to contain a single-copy transgene at a defined genomic location [21]. For *LUCL*, the segregation pattern of kanamycin resistance (conferred by *d35S::NPTII*) was consistent with the transgene being inserted into a single genomic locus. However, unlike for *LUCH*, multiple attempts to identify the insertion site in *LUCL* via thermal asymmetric interlaced PCR (TAIL-PCR) failed. This suggested that multiple copies of the transgene may be tandemly or inversely arrayed at the insertion site. To test this hypothesis, we performed Southern blot analyses on *LUCL* and *LUCH* using the *LUC* coding region as a probe. Genomic DNA from *LUCL* and *LUCH* was digested with *EcoR*I, which should release the *LUC-AP2* portion of the transgene (Figure 1A). The band corresponding to the 2.1-kb *LUC-AP2* portion was more intense in *LUCL* than in *LUCH* when the same amount of DNA was used (Figure 1C). The intensity of the band was higher than that of *LUCH* even when the amount of *LUCH* DNA was twice the amount of *LUCL* DNA (Figure 1C). Moreover, when *LUCL* genomic DNA was digested with *BamH*I, which has a single site in the transgene (Figure 1A), a band of approximately 6 kb was observed (Figure 1D, arrow). The size of this band is consistent with that of a *BamH*I fragment from two neighboring, tandemly arrayed transgenes (Figure 1A and 1D). Thus, *LUCL* is a multi-copy, single-insertion transgene.

### *LUCL* does not report miRNA activity

*LUCH* does not report miRNA activity even though it contains the miR172 binding site [21]. We wanted to know whether *LUCL*, which was derived from the same transgene in an independent transformation event, is repressed by miR172. If *LUCL* is repressed by miR172, then mutations causing reduced miR172 accumulation are expected to cause the de-repression of *LUCL*. The *dcl1-7* allele is a partial loss-of-function mutation in *DICER-LIKE1* (*DCL1*), a key factor in miRNA biogenesis [28-31]. We crossed *dcl1-7* with *LUCL* and observed luciferase luminescence in eight different F2 populations (Additional file 1: Figure S1 and data not shown). No seedlings in any of the F2 populations (Additional file 1: Figure S1) showed enhanced luciferase luminescence. We genotyped some of the seedlings and were able to identify *dcl1-7* homozygous ones. As the F2 seedlings were selected for kanamycin resistance, all contained the *LUCL* transgene, although it was not known whether they were hemizygous or homozygous for the transgene. These results suggested that *LUCL* does not report miRNA activity.

### *LUCL* is silenced by DNA methylation

**Figure 2:**
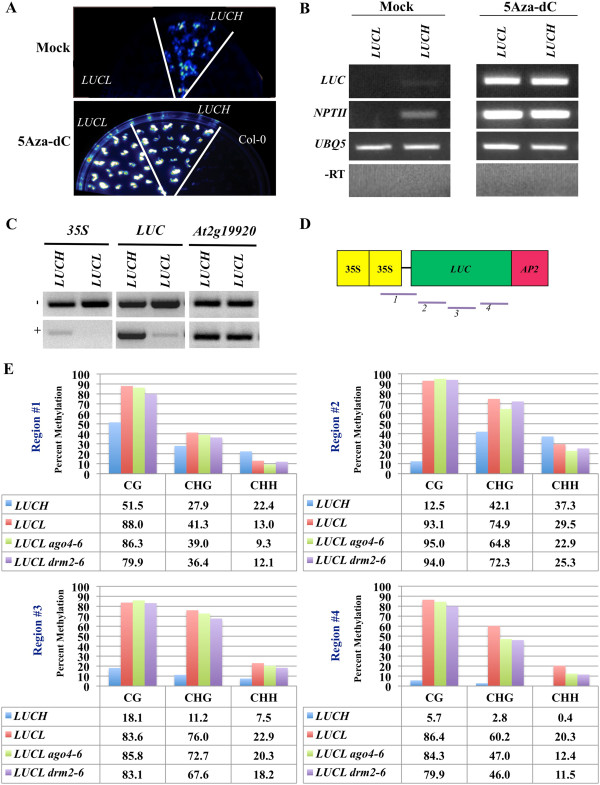
***LUCL*is silenced by DNA methylation. (A)** Effects of 5-aza-2′-deoxycytidine (5-aza-dC) treatment on *LUCH* and *LUCL*. Ten-day-old seedlings grown on plates with or without 5-aza-2′-dC were imaged for luciferase luminescence using a CCD camera. Col-0 was included as the negative control. Each blue or white spot represents a seedling. Under the same imaging conditions, 5-aza-dC-treated *LUCL* and *LUCH* seedlings had much higher levels of luciferase luminescence compared to mock (DMSO)-treated seedlings. **(B)** RT-PCR of mock-treated and 5-aza-2′-dC-treated *LUCL* and *LUCH* seedlings in (**A**). The *LUC* and *NPTII* genes are shown. *UBQ5* served as an internal loading control. ‘–RT’ indicates RT-PCR conducted in the absence of reverse transcriptase during the reverse transcription step. **(C)** Detection of DNA methylation in *LUCH* and *LUCL* by McrBC digestion of genomic DNA followed by PCR. The + gels are DNA treated with McrBC. The − gels are DNA treated in the same manner as the + gels except that no McrBC was added. *At2g19920* was used as an unmethylated internal control. **(D)** The *d35S::LUC-AP2* transgene in both *LUCH* and *LUCL*. The four lines below the rectangles mark the four regions interrogated by bisulfite sequencing in (**E**). **(E)** Detection of DNA methylation at the luciferase reporter gene in *LUCH*, *LUCL*, *LUCL ago4-6* and *LUCL drm2-6* by bisulfite sequencing. The graphs represent the percentage of DNA methylation (y-axis) at the three different cytosine contexts (x-axis). The percentage of DNA methylation is also listed in the tables below the graphs. See Additional file 1: Table S2 for bisulfite conversion rates. 5-aza-dC: 5-aza-2′-deoxycytidine; RT-PCR: reverse transcription-PCR. DMSO: Dimethyl sulfoxide; McrBC PCR: digestion of genomic DNA by McrBC followed by PCR.

Since *LUCL* is not repressed by miRNA activity, we tested whether it is repressed by DNA methylation. We grew *LUCH* and *LUCL* seedlings in a medium containing 5-aza-2′-deoxycytidine, a chemical inhibitor of DNA methyltransferase activity [32]. *LUCL* and *LUCH* seedlings treated with 5-aza-2′-deoxycytidine had higher levels of luciferase luminescence than mock-treated seedlings (Figure 2A). More importantly, the two lines had nearly equal levels of luciferase luminescence in the presence of 5-aza-2′-deoxycytidine (Figure 2A), suggesting that the lack of observable luciferase activity from *LUCL* was likely due to DNA methylation. To confirm that the observed increase in luciferase activity was due to an increase in transgene expression, we performed reverse transcription-PCR (RT-PCR) on the seedlings, as shown in Figure 2A. The expression of the *LUC* transgene as well as the nearby *NPTII* transgene was lower in *LUCL* than in *LUCH* in mock-treated seedlings (Figure 2B). The expression of both transgenes was de-repressed by treatment with 5-aza-2′-deoxycytidine (Figure 2B).

As the experiments above suggested that *LUCL* was repressed by DNA methylation, we set out to determine the levels and sequence contexts of DNA methylation as well as its distribution along the transgene in *LUCL*. We first examined the methylation status of *LUCL* by digesting genomic DNA with the restriction endonuclease McrBC followed by PCR amplification of the DNA. McrBC cuts methylated DNA in the presence of GTP [33] such that the presence of PCR products indicates lack of DNA methylation. Upon digestion of *LUCL* and *LUCH* DNA with McrBC, we found that little PCR products were observed at the *35S* region in either line (Figure 2C). This is consistent with our previous observation that the *d35S* is methylated in *LUCH*[21]. The lack of PCR products in *LUCL* suggested that the *d35S* in *LUCL* also harbors DNA methylation. In addition, the *LUC* coding region was also methylated in *LUCL*, whereas it is not in *LUCH* (Figure 2C). Therefore, *LUCH* and *LUCL* both harbor *35S* promoter methylation and *LUCL* also contains coding region methylation. We next determined the sequence contexts in which *LUCL* is methylated. We performed bisulfite sequencing of *LUCL* and *LUCH* at four regions covering the promoter and the coding region (fragments 1 to 4 in Figure 2D). Specifically, fragment 1 was from the *d35S* upstream of the *LUC* transgene (instead of the *d35S* upstream of *NPTII*) and contained 100 bp of the *LUC* coding region, and the other three fragments were from the *LUC* coding region (Figure 2D). We found that *LUCL* harbored higher levels of CG and CHG methylation and lower levels of CHH methylation at the *d35S* region relative to *LUCH* (Figure 2E, Region 1). In fact, *LUCL* exhibited high levels of CG and CHG methylation throughout the *LUC* coding region, whereas in *LUCH*, DNA methylation was restricted to the promoter and the 5′ portion of the coding region (Figure 2E, Regions 2 to 4).

### *LUCL* is repressed by *MET1*

**Figure 3:**
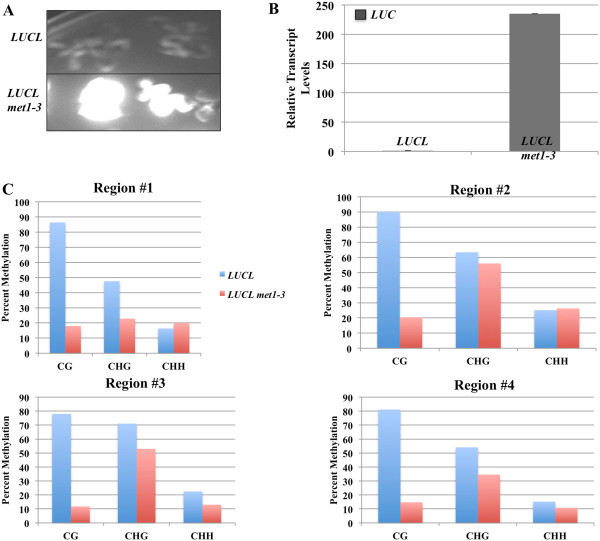
***met1-3*releases DNA methylation in*LUCL*. (A)** Luciferase luminescence of *LUCL* and *LUCL met1-3*. The top panel contains two *LUCL* seedlings and the bottom panel contains two *LUCL met1-3* seedlings. **(B)** RT-PCR of *LUC* transcript levels. *UBQ5* was used as an internal control. **(C)** Bisulfite sequencing analyses of *LUCL* (blue bars) and *LUCL met1-3* (red bars) reveal that CG methylation is reduced at all four regions tested in *LUCL met1-3*. The regions tested are indicated in Figure 2D. RT-PCR: reverse transcription-PCR.

CG maintenance methylation requires *MET1* – loss-of-function mutations in *MET1* result in global hypomethylation [2,34]. Since *LUCL* harbors high levels of CG methylation, we wanted to see whether the methylation as well as the TGS status at *LUCL* requires *MET1*. We crossed *LUCL* into *met1-3* and found that luciferase luminescence was extremely high in *LUCL met1-3* plants (Figure 3A). This was accompanied by a drastic increase in *LUC* transcript levels as determined by RT-PCR (Figure 3B). We examined the DNA methylation status in *LUCL met1-3* by bisulfite sequencing analyses at the *d35S* promoter and the *LUC* coding region. We found that that CG methylation was dramatically reduced in *LUCL met1-3* plants throughout the four regions (Figure 3C). CHH methylation was barely affected and CHG methylation was only slightly affected (Figure 3C). Taken together, the high levels of CG methylation in the promoter and gene body of *LUCL* are maintained by *MET1*, and loss of CG methylation results in strong *LUC* expression.

### *LUCL* is also repressed by RdDM

**Figure 4:**
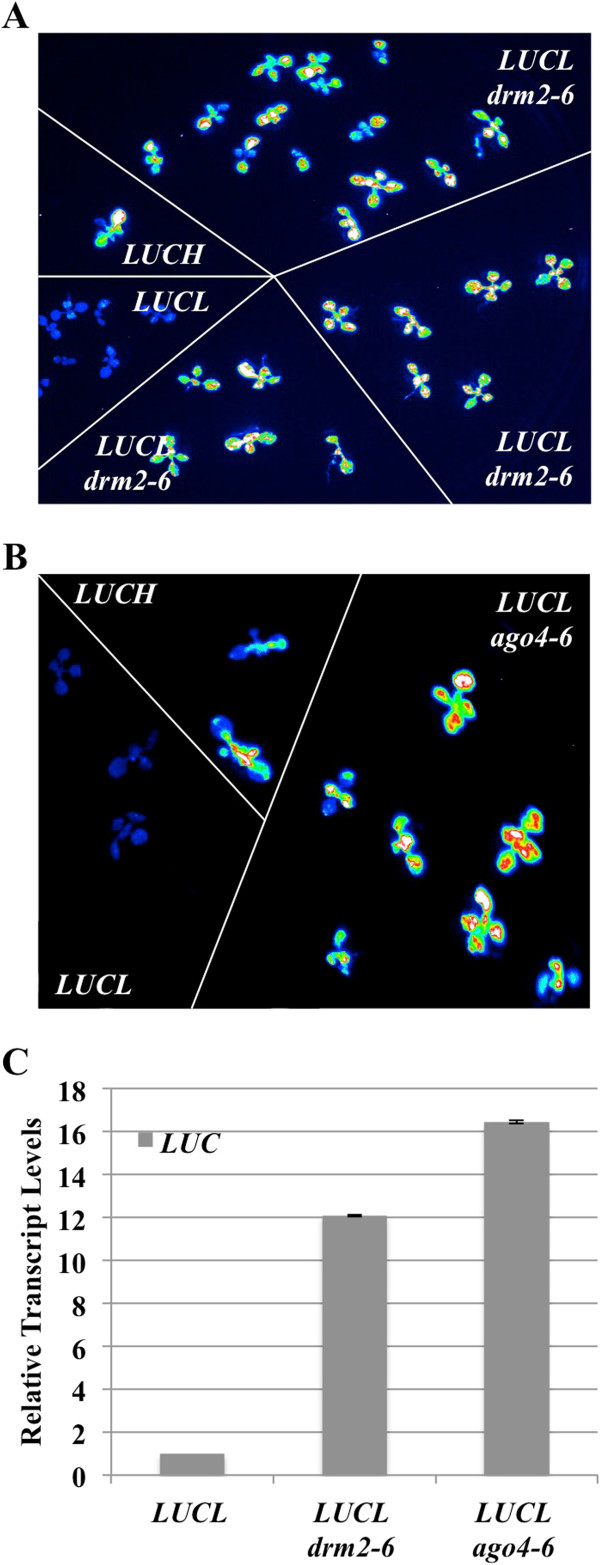
***LUCL*is weakly de-repressed by mutations in*DRM2*and*AGO4*. (A)** Luciferase luminescence of *LUCL*, *LUCH* and *drm2-6 LUCL* seedlings. **(B)** Luciferase luminescence of *LUCL*, *LUCH* and *LUCL ago4-6* seedlings. **(C)** RT-PCR of *LUC* transcript levels in *LUCL*, *LUCL drm2-6* and *LUCL ago4-6*. *UBQ5* was used as an internal control. RT-PCR: reverse transcription-PCR.

CHH methylation is maintained by RdDM involving the small RNA effector AGO4 and the *de novo* methyltransferase DRM2. Although the levels of CHH methylation in *LUCL* are relatively low (approximately 10% in the *d35S* promoter) compared to CG methylation, these levels are similar to those of CHH methylation at previously established reporter genes under the control of RdDM. For example, the *Superman* 5′ region contained 15% CHH methylation in the *clk-sk* line [35]; the *RD29A* promoter in an *RD29A::LUC* line had 6% CHH methylation in the *ros1* background in which a DNA demethylase is mutated [36]. Therefore, it is also possible that *LUCL* is repressed by RdDM. To test this, we crossed *LUCL* with *drm2-6* and *ago4-6*, mutations in *DRM2* and *AGO4*, respectively. These alleles were previously isolated in our lab and found to de-repress *LUC* expression from *LUCH*[21]. *LUCL drm2-6* and *LUCL ago4-6* plants had higher levels of luciferase luminescence than *LUCL* plants (Figure 4A and 4B). RT-PCR showed that *LUCL drm2-6* and *LUCL ago4-6* plants had higher levels of *LUC* transcripts (Figure 4C), but the extent of *LUC* de-repression in *drm2-6* or *ago4-6* was much lower than that in *met1-3* (compare Figure 4C to Figure 3B). We performed bisulfite sequencing in *LUCL*, *LUCL drm2-6* and *LUCL ago4-6* to determine the effects of the *drm2* and *ago4* mutations on DNA methylation at the transgene. Little difference in CG or CHG methylation could be detected at the *d35S* promoter or in the *LUC* coding region in the two mutants compared to wild type (Figure 2E). For CHH methylation, only the 3′ portion of the *LUC* coding region showed an approximately 50% reduction in the two mutants (Figure 2E). We conclude that *LUCL* is a sensitive reporter such that even a small reduction in DNA methylation is reflected by moderate de-repression of the reporter.

### A chemical screen confirms that *LUCL* reports DNA methylation

**Figure 5:**
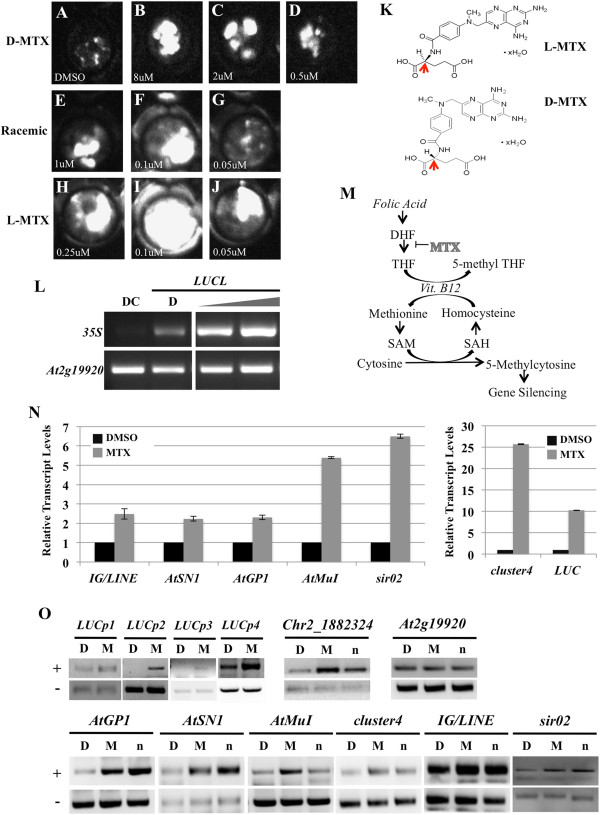
**MTX releases DNA methylation of*LUCL.*(A-J)** Luciferase luminescence of *LUCL* seedlings treated with various compounds. **(A)** DMSO-treated *LUCL* seedlings. **(B-D)** D-MTX-treated *LUCL* seedlings. **(E-G)***LUCL* treated with a mixture of D- and L-MTX. **(H-J)** L-MTX-treated *LUCL* seedlings. The concentrations of the chemicals are as indicated in **(B-J)**. **(K)** Chemical structures of L-MTX (top) and D-MTX (bottom). The arrows indicate the position of chirality of the two forms. **(L)** McrBC-PCR-based methylation assay of *LUCL* seedlings treated with D-MTX. DC: DMSO-treated Col-0 control, D: DMSO-treated *LUCL*. The gray triangle represents increasing concentrations of MTX (2 μM for the left lane and 8 μM for the right lane). **(M)** MTX inhibits SAM biosynthesis to indirectly affect gene silencing via DNA methylation. MTX inhibits the conversion of DHF to THF. Under normal circumstances, the energy given off by the conversion of THF to 5-methyl THF promotes the production of methionine from homocysteine and vitamin B12. **(N)** Expression of *LUCL* and six endogenous RdDM loci in DMSO (control)- and MTX-treated seedlings as determined by RT-PCR. **(O)** McrBC-PCR-based methylation assay of *LUCL* seedlings treated with DMSO (D) or MTX (M), and non-treated *nrpe1-11* seedlings (n). Two biological replicates gave similar results and only one is shown here. +: McrBC digested; -: non-digested. The six loci in the bottom panel are known to undergo RdDM. *LUCp1* to *LUCp4* correspond to regions 1 to 4 of the *LUCL* transgene in Figure 2D. Chr2_1882324 is a region that harbors DNA methylation in wild type. *At2g19920* is a gene that does not harbor any DNA methylation and is used as an internal loading control. DHF: dihydrofolate; DMSO: dimethyl sulfoxide; McrBC-PCR: digestion of genomic DNA by McrBC followed by PCR; MTX: methotrexate; RT-PCR: reverse transcription-PCR; SAH: S-adenosylhomocysteine; SAM: S-adenosyl methionine; THF: tetrahydrofolate.

Since *LUCL* is silenced by DNA methylation, we reasoned that we could use luciferase luminescence as a readout to identify chemical compounds that affect DNA methylation. We screened 24,970 chemical compounds against *LUCL* seedlings at the two-leaf stage. One of the hits, methotrexate (MTX), released luciferase activity in a dose-dependent manner (Figure 5A, B, C, D). MTX is a compound that inhibits dihydrofolate reductase (DHFR), an enzyme that participates in tetrahydrofolate (THF) synthesis. DHFR catalyzes the conversion of dihydrofolate (DHF) to THF [37] (Figure 5M). The energy given off by the conversion of THF to 5-methyl THF catalyzes the production of methionine from homocysteine and vitamin B12. Therefore, MTX ultimately prevents the production of the methyl donor, S-adenosyl methionine (SAM) [20] (Figure 5M). MTX is found in two forms, D and L (in reference to the molecule’s chirality) (Figure 5K, arrows). While we tried to perform the secondary validations with the compound, we found that the compound pulled from the initial screen possessed D chirality (Figure 5K, bottom), and the vendor discontinued the product. Thus, we tested *LUCL* with L-MTX and a racemic mixture of D- and L-MTX. Both L-MTX and the racemic mixture were able to release luciferase activity of *LUCL* at concentrations lower than that of D-MTX (Figure 5E, F, G, H, I, J). L-MTX is more efficiently taken up by human cells than D-MTX [38]; perhaps this is also true in plants. We tested whether MTX released DNA methylation at *LUCL* by McrBC-PCR. Indeed, we found that D-MTX released DNA methylation at the *d35S* promoter in a concentration-dependent manner (Figure 5L).

Next, we examined whether MTX affects DNA methylation and/or transcriptional silencing of endogenous loci. Seedlings were treated with DMSO (control) or a racemic mixture of MTX, and the expression of the luciferase transgene as well as six endogenous loci known to undergo RdDM was determined by RT-PCR. MTX led to the de-repression of the luciferase transgene and the six endogenous loci (Figure 5N). The DNA methylation status of the six loci, as well as Chr2_1882324 (another locus that harbors DNA methylation) and the luciferase transgene, was evaluated by McrBC-PCR. In addition to the *d35S* promoter, the luciferase coding region showed reduced DNA methylation in MTX-treated seedlings (Figure 5O). MTX treatment also led to reduced DNA methylation at the six endogenous loci (Figure 5O). The effect of MTX was similar to that of the *nrpe1* mutation (in the largest subunit of Pol V) in the reduction of DNA methylation at these loci (Figure 5O).

## Conclusions

We developed a luciferase-based reporter transgene (*LUCL*) that reports TGS by *MET1*-mediated CG methylation as well as *de novo* methylation by RdDM. Like existing TGS reporter systems [2,39], *LUCL* is suitable for identifying positive players involved in *de novo* methylation by RdDM and CG maintenance methylation. The lack of luciferase luminescence from *LUCL* allows for facile genetic or chemical screens in which mutations or compounds that release DNA methylation could be easily identified based on the appearance of luciferase luminescence. Using this reporter line, we have screened approximately 25,000 small molecules and obtained two reproducible hits. One of these hits, MTX, serves as a proof-of-concept as its negative function in methyl biogenesis is known [20]. Another feature of this reporter system is that it harbors high levels of DNA methylation in the *LUC* coding region. Thus, *LUCL* may be used as a probe to dissect the molecular mechanism and function of gene body methylation.

## Methods

### Plant material

*Arabidopsis* mutants used in this study, *rdr6-11*[25], *dcl1-7*[29], *met1-3*[40], *drm2-6*[21], *ago4-6*[21] and *nrpe1-11 *[41,42], are in the Col-0 background.

### Growth conditions and luciferase live imaging

*Arabidopsis thaliana* seeds were surface-sterilized with 30% bleach, planted on Murashige and Skoog (MS) agar plates containing kanamycin (30 mg/mL for lines containing *LUCL*) and stratified at 4°C for 2 days. Seedlings were grown at 23°C under continuous light for 10 days. All experiments with *LUCL* and *LUCH* were performed with 10-day-old seedlings. For the chemical screen, two seeds were plated into each well in a 96-well plate. After 7 days, chemicals were added in each well, except for the first column, in which DMSO was added as a negative control. Three days later, the plates were imaged for luciferase activity [21]. For the secondary screening of MTX, D- and/or L-MTX (Sigma) were added individually per well. After images were taken, plants were collected for subsequent methylation assays. For luciferase live imaging, 1 mM luciferin (Promega) in 0.01% Triton X-100 was sprayed onto the seedlings, which were incubated in the dark for 5 min before images were taken. Luciferase images were taken using a Stanford Photonics Onyx Luminescence Dark Box with a Roper Pixis 1024B camera at the UC Riverside Genomics Core Facility.

### Construction of transgene and Southern blot analysis

*LUCL* and *LUCH* are two independent transgenic lines containing the same transgene, which has been previously described [21]. Southern blot analysis was performed according to the standard protocol [43] to evaluate the copy number of *LUCL* using the full-length *LUC* coding region as the probe. The probe was amplified with primers lucp6 and lucp7, and radiolabeled with the RPN1633 Rediprime II random prime labeling system (GE Healthcare). Primers used were previously described and are listed in [21] and Additional file 1: Table S1.

### Analysis of DNA cytosine methylation

For the McrBC-PCR assay, two reactions were set up for each genomic DNA sample: McrBC treated and untreated. Next 400 ng genomic DNA was digested with McrBC (New England Biolabs) for 30 min at 37°C in a 20 μl reaction. Then 1 μl of restricted genomic DNA was used as the template and genomic regions corresponding to the *LUCL* transgene or endogenous loci were amplified. *At2g19920* was used as a loading control. See Additional file 1: Table S1 for sequences of primers.

For bisulfite sequencing, in Figure 2, 1 μg of RNase-treated genomic DNA was subjected to bisulfite conversion using the EpiTect Bisulfite Kit per the manufacturer’s instructions (Qiagen). For Figure 3, 400 ng of RNase-treated genomic DNA derived from leaf tissue from *LUCL* and *LUCL met1-3* plants was subjected to bisulfite conversion using the MethylCode™ Bisulfite Conversion Kit per the manufacturer’s instructions (Invitrogen). The PCR reactions with primers YZ 35S Bis F and YZ LUC Bis R as well as another three sets of primers that covered the *LUC* coding region were performed using the converted DNA as a template as described previously [21], purified via gel extraction per the manufacturer’s instructions (Qiagen or Zymo), and cloned into the pGEM-T Easy vector (Promega). A minimum of 24 clones were sequenced for each sample and unique clones were analyzed for DNA methylation with Kismeth [44,45]. To determine the conversion efficiency, PCR reactions were conducted with primers specific for chloroplast DNA using the same converted DNA as above and the PCR products were processed in the same manner. At least 15 unique clones were selected for analysis by Kismeth. As chloroplast DNA is unmethylated, conversion efficiency could be determined. See Additional file 1: Table S2 for conversion rates of various samples. For 5-aza-2′-deoxycytidine (Sigma) treatment, seeds were germinated and grown on an MS agar medium containing 1% sucrose and 7 μg/mL of the chemical for 2 weeks and luciferase images were taken.

### RT-PCR

RNA was isolated with Tri-reagent (Molecular Research Center) from 10-day-old seedlings from *LUCL met1-3*, *LUCL ago4-6* and *LUCL drm2-6* plants as previously described [46]. For the RT-PCR in Figures 3 and 4, older leaf tissue from *LUCL met1-3*, *LUCL ago4-6*, and *LUCL drm2-6* plants was utilized. For the RT-PCR in Figure 5, 10-day-old, chemical-treated seedlings were used. cDNA was synthesized from 5 μg (14 μg for Figure 5) of DNaseI (Invitrogen)-treated total RNA using reverse transcriptase (Fermentas) and oligo-dT (Fermentas) as previously described [21]. The sequences of primers are listed in Additional file 1: Table S1.

### Chemical screening

Small molecule compounds used for the chemical screen consist of: 1,200 from LifeSciences, 2,000 from Spectrum and 400 from Myria/Sigma from the UCR small compounds collection [47]; 4,204 from a triazine-tagged library [48,49]; 2,768 from Clickables [50] and 3,580 from LATCA [51]. The screening was performed at the Chemical Screening Facilities at UC Riverside.

## Abbreviations

5-aza-2′-dC: 5-aza-2′-deoxycytidine; bp: base pair; DHF: dihydrofolate; DHFR: dihydrofolate reductase; DMSO: Dimethyl sulfoxide; GTP: guanosine triphosphate; miRNA: microRNA; MTX: methotrexate (also known as amethopterin); nt: nucleotide; PCR: polymerase chain reaction; RdDM: RNA-directed DNA methylation; RT-PCR: reverse transcription-PCR; SAH: S-adenosylhomocysteine; SAM: S-adenosyl methionine; siRNA: small interfering RNA; ssRNA: single-stranded RNA; TAIL-PCR: thermal asymmetric interlaced PCR; TGS: transcriptional gene silencing; THF: tetrahydrofolate

## Competing interests

The authors declare that they have no competing interests.

## Authors’ contributions

TTD and XC wrote the manuscript. TTD designed the chemical screen and performed genetic analyses of *LUCL*, Southern blot analyses, bisulfite sequencing analyses, luciferase imaging, RT-PCR and McrBC analysis. TTD and MOL performed the chemical screen. MOL performed the luciferase imaging for the MTX-treated seedlings. SYW did the 5-aza-2′-dC treatment and RT-PCR of treated seedlings. XL, TTD and LA generated the *LUCL met1-3*, *LUCL ago4-6* and *LUCL drm2-6* genotypes and analyzed them. SL performed the McrBC analysis on *LUCH* and *LUCL*. AD and SRC provided chemicals, performed small molecule perturbations and offered intellectual assistance with regards to the chemical screen. BZ transformed *LUCL* into *rdr6-11*. XC constructed the reporter plasmid, conceived and guided the project. All authors read and approved the final manuscript.

## Supplementary Material

Additional file 1: Figure S1 *LUCL* is not regulated by the miRNA pathway. Luciferase luminescence of *LUCL*, *LUCH*, and seedlings from several F2 populations (#101, 103 and 104) of *dcl1-7* crossed to *LUCL*. In the F2 population, none of the seedlings showed de-repression of luciferase activity. **Table S1.** DNA oligonucleotides used in this study. **Table S2.** Conversion rates for the bisulfite sequencing experiments. Click here for file
