# Supplementary material for: Generation of a luciferase-based reporter for CHH and CG DNA methylation in Arabidopsis thaliana
Source: Silence. 2013 Apr 5;4:1. doi: 10.1186/1758-907X-4-1 (PMC3623655; doi:10.1186/1758-907X-4-1)
Supplement: Additional file 1: Figure S1 — LUCL is not regulated by the miRNA pathway. Luciferase luminescence of LUCL, LUCH, and seedlings from several F2 populations (#101, 103 and 104) of dcl1-7 crossed to LUCL. In the F2 population, none of the seedlings showed de-repression of luciferase activity. Table S1. DNA oligonucleotides used in this study. Table S2. Conversion rates for the bisulfite sequencing experiments. [file 1758-907X-4-1-S1.pdf]

Additional files

Additional file 1

Figure S1. *LUCL* is not regulated by the miRNA pathway. (A) Luciferase luminescence of *LUCL*, *LUCH,* and seedlings from several F2 populations (#101, 103, and 104) of *dcl1-7* crossed to *LUCL*. In the F2 population, none of the seedlings showed de-repression of luciferase activity.

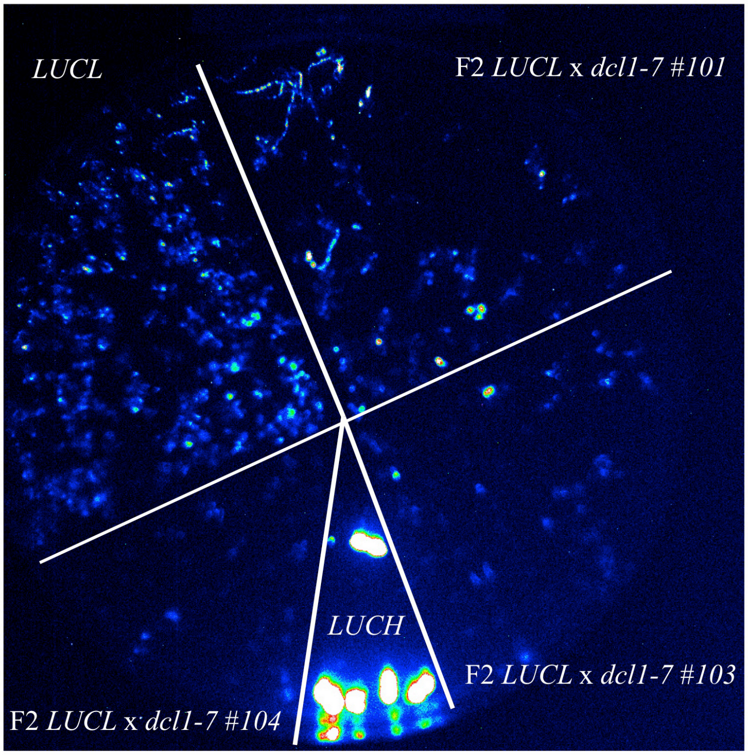

Additional File 2. Supplementary Tables

Table S1. DNA oligonucleotides used in this study

Name	Sequence	Purpose
lucp6	GCACCCGGGGAAGACGCCAAAAACATAAA AGAAA	McrBC-PCR, Southern blot
lucp7	GGACCCGGGTGCGATCTTTCCGCCCTTCTTG GCCT	McrBC-PCR, Southern blot
Actin1-F	CCAAGCAGCATGAAGATCAA	McrBC-PCR
Actin1-R	TGAACAATCGATGGACCTGA	McrBC-PCR
35Sf	CAAAGCAAGTGGATTGATGTGA	McrBC-PCR
35Sr	TTTCCACGATGCTCCTCGT	McrBC-PCR
At2g19920-F	TCACCCGAACAGTTGGAAGAA	McrBC-PCR
At2g19920-F	GTGAGGAACCGGTCCATTATTGCT	McrBC-PCR
YZ 35S Bis F	AttAtTGTyGGtAGAGGtATtTTGAAyGATAGtt	Bisulfite sequencing region 1
YZ LUC Bis R	CATCTaTAAAAaCAATTaTTCCAaaAACCAaa	Bisulfite sequencing region 1
BS-LUC CDS F1	AYTGYATAAGGYTATGAAGAGAT	Bisulfite sequencing region 2
BS-LUC CDS R1	CACAAAATCRTATTCATTAAAACC	Bisulfite sequencing region 2
BS-LUC CDS F2	GGTTTTAATGAATAYGATTTTGTG	Bisulfite sequencing region 3

BS-LUC CDS R2	ACCTCTTRRCAACCRCTTCCC	Bisulfite sequencing region 3
BS-LUC CDS F3	TGAAGYGAAGGTTGTGGATYTGG	Bisulfite sequencing region 4
BS-LUC CDS R3	CRATCTTTCCRCCCTTCTT	Bisulfite sequencing region 4
N_UBQ5	GGTGCTAAGAAGAGGAAGAAT	RT-PCR, loading control
C_UBQ5	CTCCTTCTTTCTGGTAAACGT	RT-PCR, loading control
Kan-RT-F	AGGTTCCATCTGCCAGGTATCA	RT-PCR for NPTII
Kan-RT-R	CCCGGTATCCAGATCCACAA	RT-PCR for NPTII
AtSN1-F	ACCAACGTGCTGTTGGCCCAGTGGTAAATC	Real-time RT-PCR/McrBC
AtSN1-F	ACCAACGTGCTGTTGGCCCAGTGGTAAATC	Real-time RT-PCR/McrBC
AtGP1-F	TGGTTTTTCCTGTCCAGTTTG	Real-time RT-PCR/McrBC
AtGP1-R	AACAATCCTAACCGGGTTCC	Real-time RT-PCR/McrBC
IG/LINE-F	AACTAACGTCATTACATACACATCTTG	Real-time RT-PCR/McrBC
IG/LINE-R	AATTAGGATCTTGTTTGCCAGCTA	Real-time RT-PCR/McrBC
AtMuI-F	CCGAGAACTGGTTGTGGTTT	Real-time RT-PCR/McrBC

AtMuI-R	GCTCTTGCTTTGGTGATGGT	Real-time RT-PCR/McrBC
Cluster4-F	CGTCCTCAAAGTTCCAGA	Real-time RT-PCR/McrBC
Cluster4-R	GGTATTCTCCATCCCAAAG	Real-time RT-PCR/McrBC
LUCM5-F	CTCCCCTCTCTAAGGAAGTCG	Real-time RT-PCR
LUCR5-F	CCAGAATGTAGCCATCCATC	Real-time RT-PCR
IR71-F	TATCATCCTTCTGGTTTTGG	Real-time RT-PCR
IR71-R	AAGCAACATTCATTTCAGC	Real-time RT-PCR
Chr2_1882324	CGAGCCAAAAATTGTTGAAAT	McrBC
Chr2_1882324	TTTGAGGGGATAGAGTATCTCTTG	McrBC

Table S2. Conversion rates for the bisulfite sequencing experiments

**Genotype**	**CG Methylation**	**CHG Methylation**	**CHH Methylation**
LUCH	100%	99.4%	99.5%
LUCL	100%	100%	99.5%
LUCL met1-3	99.3%	100%	99.5%
LUCL	99.2%	100%	99.3%
LUCL ago4-6	99.1%	100%	99.9%
LUCL drm2-6	100%	100%	99.9%
